# Metagenomic next-generation sequencing-based characterization of the viral spectrum in clinical pulmonary and peripheral blood samples of patients

**DOI:** 10.3389/fcimb.2025.1562965

**Published:** 2025-07-22

**Authors:** Ning Zheng, Hai-Long Yu, Bing-Jie Zhang, Dan Wang, Ya-Liang Ji, Lu-Lu Dai, Wen Li, Sheng-Hui Li, Zhi-Liang Hu, Yi-Shan Zheng

**Affiliations:** ^1^ Department of Critical Care Medicine, The Second Hospital of Nanjing, Affiliated to Nanjing University of Chinese Medicine, Nanjing, China; ^2^ Puensum Genetech Institute, Wuhan, China; ^3^ Department of Infectious Disease, The Second Hospital of Nanjing, Nanjing University of Chinese Medicine, Nanjing, China; ^4^ Center for Global Health, School of Public Health, Nanjing Medical University, Nanjing, China

**Keywords:** metagenomic next-generation sequencing, viral composition, bronchoalveolar lavage fluid, peripheral blood, virus-bacteria interactions

## Abstract

**Background:**

Metagenomic next-generation sequencing (mNGS) enables comprehensive profiling of viral communities in clinical samples. However, comparative analyses of the virome across anatomical compartments and disease states remain limited. This study aims to characterize the virome in bronchoalveolar lavage fluid (BALF) and peripheral blood samples from patients with various clinical conditions using mNGS.

**Methods:**

A total of 338 clinical samples—including 240 BALF and 69 blood samples for DNA sequencing, and 18 BALF and 11 blood samples for RNA sequencing—underwent shotgun metagenomic sequencing. Following removal of host-derived reads, high-quality non-human sequences were aligned to a viral reference database. Virome composition was assessed through alpha and beta diversity metrics. Principal coordinates analysis was used to evaluate disease-related variation, and virus–bacteria associations in BALF were investigated via Spearman correlation.

**Results:**

Sequencing yielded an average of 51 million raw reads per sample, resulting in approximately 8 million non-human reads after host filtering. Distinct virome profiles were observed between BALF and blood samples. Bacteriophages dominated all groups, with *Siphoviridae* and *Myoviridae* as the most abundant families, although only 13.6% of viral abundance could be assigned to known families. Diversity analyses revealed significant differences between BALF and peripheral blood, and DNA-sequenced BALF samples showed disease-specific viral signatures in pulmonary infections. In contrast, tumor presence had no significant effect on virome composition in either BALF or blood. Network analysis identified complex virus–bacteria correlations in BALF, with genera such as *Haemophilus*, *Megasphaera*, and *Treponema* as key bacterial hosts.

**Conclusions:**

This study reveals pronounced differences in virome composition between the respiratory and circulatory systems and highlights the specific influence of pulmonary disease—but not tumors—on the pulmonary virome. The observed virus–bacteria networks provide novel insights into pulmonary microbial ecology and underscore the importance of integrating host and disease context in virome studies.

## Introduction

Recent advances in metagenomic next-generation sequencing (mNGS) have transformed viral diagnostics and research by enabling the unbiased detection and comprehensive profiling of viral communities in diverse clinical samples (Dhariwal et al., 2017; Mehta et al., 2019; Deng et al., 2020). Traditional diagnostic methods often lack the sensitivity and breadth needed to detect a wide range of viral pathogens, especially in complex clinical samples like bronchoalveolar lavage fluid (BALF) and peripheral blood (Kitsios, 2018; Zhu et al., 2018), In contrast, mNGS offers high-depth sequencing that enables comprehensive detection and characterization of known, novel, and rare viruses in a single, unbiased assay (Väisänen et al., 2018; Kwok et al., 2020).

Among clinical specimens, BALF and peripheral blood are two critical biological compartments for viral detection. BALF, obtained from the lower respiratory tract, plays a pivotal role in diagnosing respiratory infections such as pneumonia and tuberculosis (Sun et al., 2018; Walter et al., 2019; Cao et al., 2020; Raghav et al., 2020; Ray et al., 2020). It has been successfully used to identify a range of respiratory viruses—including influenza, respiratory syncytial virus (RSV), and adenovirus—informing treatment decisions and improving clinical outcomes (Sumino et al., 2010; Lee et al., 2016; Bao et al., 2022). In contrast, peripheral blood reflects systemic viral dissemination, and is commonly used to detect circulating pathogens such as cytomegalovirus (CMV), Epstein-Barr virus (EBV), and HIV (Razonable et al., 2002; Kessler et al., 2008; Guo et al., 2020). Blood-based virome studies have revealed rich viral diversity (e.g., DNA and RNA viruses) and provided insights into host immune status, especially in immunocompromised patients (Li et al., 2013).

Understanding the viral landscape of bronchoalveolar lavage fluid (BALF) and peripheral blood is essential for improving the diagnosis and treatment of infectious diseases. In patients with severe respiratory infections, identifying viral pathogens in BALF can help distinguish between viral and bacterial etiologies, thereby supporting targeted antimicrobial therapy and reducing unnecessary antibiotic use (Vena et al., 2020). Similarly, profiling the blood virome, particularly in immunocompromised individuals, can facilitate the early detection of opportunistic viral infections, enabling timely intervention and improved clinical outcomes (Van Rijn et al., 2019).

In this study, we performed metagenomic shotgun sequencing on 338 clinical samples, including 240 BALF and 69 peripheral blood samples for DNA sequencing, as well as 18 BALF and 11 peripheral blood samples for RNA sequencing. Our objectives were to (i) compare viral diversity and taxonomic composition between BALF and peripheral blood; (ii) investigate differences in virome profiles derived from DNA and RNA sequencing; and (iii) evaluate the influence of disease context on virome structure.

## Methods

### Subject and sample collection

We retrospectively collected 338 samples from patients with confirmed infections who were admitted to the Second Hospital of Nanjing, Jiangsu, China, between May 2023 and October 2023. Samples were included if mNGS had been performed on BALF or peripheral blood. Clinical data were collected for each sample. Medical records were reviewed to obtain baseline information, including age, sex, diagnosis, and clinical outcome (**Supplementary Table 1**). Informed consent was obtained from all participants.

### BALF samples and peripheral blood samples for DNA and RNA extraction

Blood samples (3 mL) and BALF samples (1.5–3 mL) were drawn from patients. The blood samples were stored at room temperature for 3–5 minutes and centrifuged at 4,000 rpm for 10 min at 4°C within 8 h of collection. The plasma was then aspirated and transferred to a sterile centrifuge tube for further use. BALF samples were processed as follows: Saponin was added to 0.45mL sample at a final concentration of 0.025%. Then the sample was fully vortexed for 15s and incubated for 5 min at 25 °C. 75 μL was added for dehosting process. The sample was fully vortexed for 15s and incubated at 37°C for 10 min. Then the sample was centrifuged at 18,000 g for 5 min and ~70-80μL were remained at the bottom after removal of 450μL supernatant. 800μL PBS was added to the tube and fully vortexed. After centrifugation at 18,000 g for 5 minutes, 800μL supernatant was discarded and ~ 70-80μL were remained at the bottom. Add 370μL TE-buffer to the tube, followed by shaking. Then 7.2 μL lysozyme was added for wall-breaking reaction. 250 μL 0.5 mm glass bead were attached to a horizontal platform on a vortex mixer and agitated vigorously at 2800–3200 rpm for 30 min. 0.3mL sample was separated into a new 1.5 mL microcentrifuge tube. For both peripheral blood samples and BALF samples, DNA extraction was performed using the TIANamp Micro DNA Kit (DP316, TIANGEN Biotech, Beijing, China) according to the manufacturer’s instructions. The extracted DNA was then used for constructing DNA libraries.

Blood samples (3 mL) and BALF samples (1.5–3 mL) were drawn from patients. The blood samples were centrifuged at 2910 rcf at 4°C for 10 minutes within 8 hours. Then 300 μL of plasma was collected and stored for further processing. BALF samples were processed as follows: If the sample was found to be thick, 450 µL of sample was taken with a 1 mL broad nozzle (if the sample was too thick to take 450 µL of sample, supplemented with normal saline) and added to a 2.0 mL centrifuge tube. At the same time, 7.5 μL LDTT solution (2M) was added to the sample, vortexed and mixed for 15 s. Then, the sample was centrifuged briefly and maintained at 30 °C for 10 min. After centrifugation at 8000 rcf for 30 s, 300 μL of supernatant was collected and stored for further processing. RNA extraction was performed on both the prepared plasma sample (300 μL) and the BALF supernatant sample (300 μL) using the Tiangen Magnetic Bead-based Pathogen Microorganism DNA/RNA Extraction Kit (Catalog No. NG550-01), strictly according to the manufacturer’s instructions.

### DNA and RNA sequencing

DNA libraries were constructed through fragmentation, end-repair, adapter-ligation, and PCR amplification, while RNA-derived libraries involved fragmentation of double-stranded cDNA followed by identical end-repair, adapter-ligation, and PCR steps. All libraries underwent unified quality control: fragment size (~300 bp) was verified using Agilent 2100 Bioanalyzer, and concentration was quantified with Qubit dsDNA HS Assay Kit. Qualified libraries were pooled equimolarly, circularized into single-stranded loops, and amplified via rolling circle replication (RCA) to generate DNA Nanoballs (DNBs). DNBs were loaded onto BGISEQ-50/MGISEQ-2000 sequencing chips for high-throughput sequencing.

### Preprocessing of metagenomic datasets and viral database

To ensure data quality. The raw reads form each sample were filtered using fastp v0.23.4 (Chen et al., 2018) with the options ‘-w 8 -q 20 -u 30 -n 5 -y -Y 30 –trim_poly_g –trim_poly_x -l 40’ to remove low-quality reads. Subsequently, the filtered reads were mapped to the GRCh38 reference genome using bowtie2 v2.4.4 (Langmead and Salzberg, 2012) with the parameters ‘-end-to-end –no-head -p 8 –mm -fastp’ to remove human reads. The remaining reads were considered clean reads for each sample.

To analyze the composition of the BALF and peripheral blood viral community in metagenomic samples, we used a virus database comprising over 65,000 nonredundant viral operational taxonomic units (vOTUs) as the reference. Firstly, we downloaded the virus databases of NCBI, Oral Virus Database (OVD) (Li et al., 2022) and Human Virus Database (HVD) (Ye et al., 2022). Then, the viral genomes were clustered into a vOTU based on a nucleotide similarity threshold of 95% across 85% of the genome using BLASTn v2.12.0 with the parameters ‘-evalue 1e-10 -word_size 20’. The representative genome was identified as the largest viral sequence in each vOTU, leading to the creation of a dereplicated virus database.

### Taxonomic profiling of the virome

To generate taxonomic profiles of pulmonary and peripheral blood samples, the clean reads in each sample were mapped to virus database using bowtie2 v2.4.4 with options ‘–end-to-end –fast –no-unal’, The relative abundance of each vOTU was calculated as the proportion of reads mapped to the vOTU to the total number of reads mapped to any vOTU in each metagenome. Furthermore, the relative abundance of each viral family was determined by aggregating the relative abundances of vOTUs annotated with the corresponding family.

### Statistical analyses

Statistical analysis and visualization were implemented in R v4.3.1. BALF and peripheral blood virome diversity was estimated based on the profiles at the vOTU level. Simpson and Shannon diversity indices were measured by the diversity function in the vegan package. Bray-Curtis distances between samples were measured by the vegdist function in the vegan package. Principal coordinate analysis (PCoA) of Bray-Curtis distances was carried out via the pcoa function in the ape package. Permutational multivariate analysis of variance (PERMANOVA) was carried out using the adonis function in the vegan package.

## Results

### Overview of viral composition in BALF and peripheral blood samples

**Figure 1:**
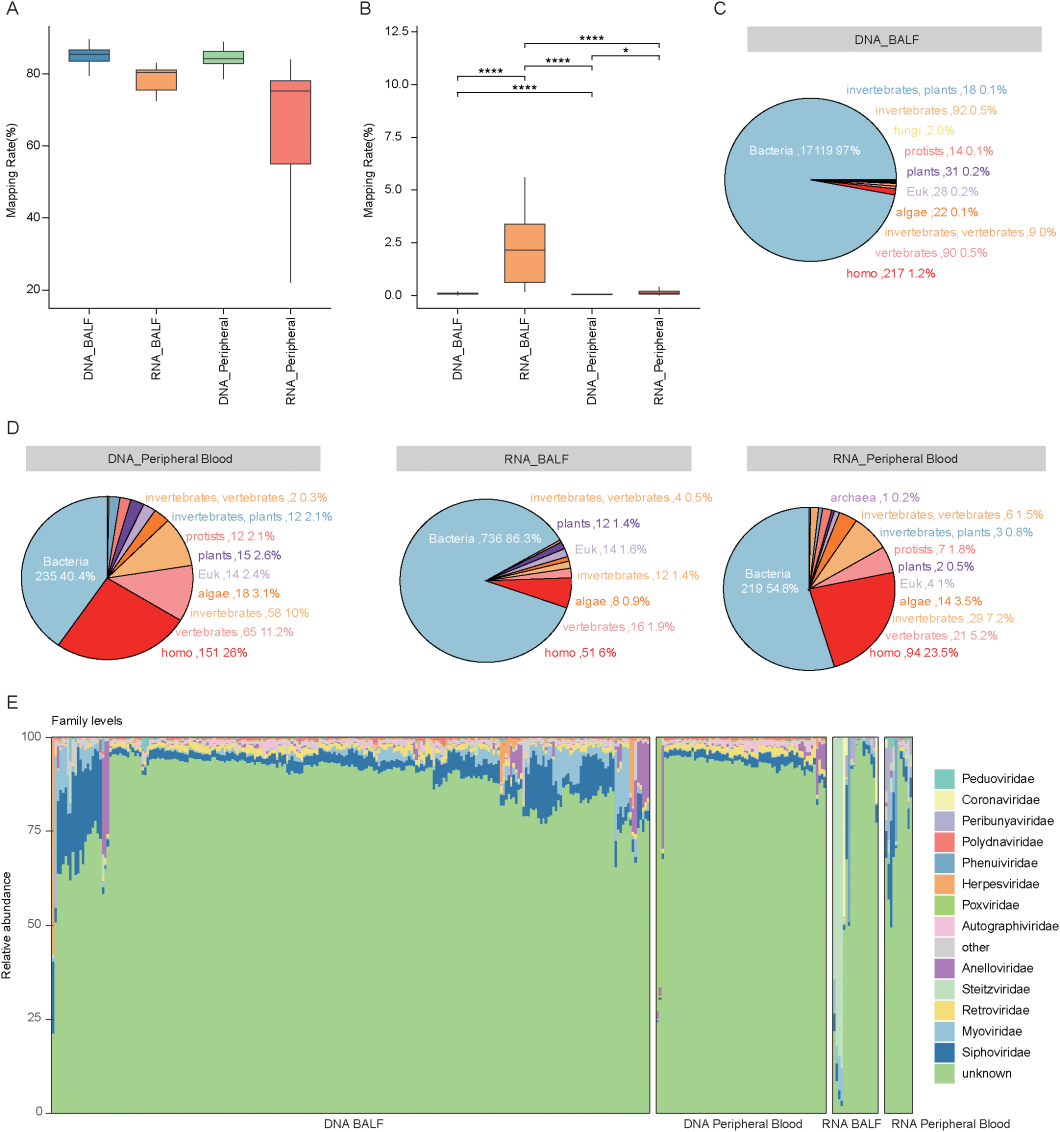
Analysis of viral composition in BALF and peripheral blood samples using DNA and RNA sequencing. **(A)** Box plots showing the mapping rates of raw reads to the human reference genome for DNA and RNA samples from BALF and peripheral blood. The mapping rate represents the percentage of raw reads that aligned to the human genome. **(B)** Box plots showing the mapping rates of clean reads to the virus database (*P < 0.05, ****P < 0.0001). **(C, D)** The pie chart shows the number and proportion of viruses from different host sources in BALF and peripheral blood samples based on the results of DNA and RNA sequencing. Different colors in the chart represent different host sources. **(E)** Bar graph showing viral composition at the family level in BALF and peripheral blood samples.

Shotgun metagenomic sequencing of 338 clinical samples yielded an average of 51 million raw reads and 2.59 Gbp per sample. These samples consisted of 240 BALF samples (231 from pulmonary infection and 51 from tumor) and 69 peripheral blood samples (41 from pulmonary infection and 27 from tumor) for DNA sequencing, as well as 18 BALF samples (18 from pulmonary infection and 5 from tumor) and 11 peripheral blood samples (10 from pulmonary infection and 4 from tumor) for RNA sequencing. To eliminate host contamination, raw reads were mapped to the human reference genome, with an average alignment rate of 82.7% across all samples, indicating a high proportion of host-derived sequences (**Figure 1A**). After host sequence removal, approximately 8 million high-quality non-human reads were retained for downstream analysis. These reads were aligned to a viral reference database containing over 65,000 high-completeness dereplicated vOTUs. Among the four sample groups, the RNA-BALF group exhibited the highest viral mapping rate, and sequencing modality significantly influenced mapping efficiency even within the same sample type (**Figure 1B**). Notably, our findings indicated that viruses in all groups predominantly originated from bacteria, with proportions of 97.0% (n = 17,119) in the DNA-BALF group, 40.4% (n = 235) in the DNA-peripheral-blood group, 86.3% (n = 736) in the RNA-BALF group, and 54.8% (n = 219) in the RNA-peripheral-blood group. Homo was identified as the next most significant source, accounting for 1.2% (n = 217), 26% (n = 151), 6% (n = 51), and 23.5% (n = 94) in the respective groups (**Figures 1C, D**).

**Figure 2:**
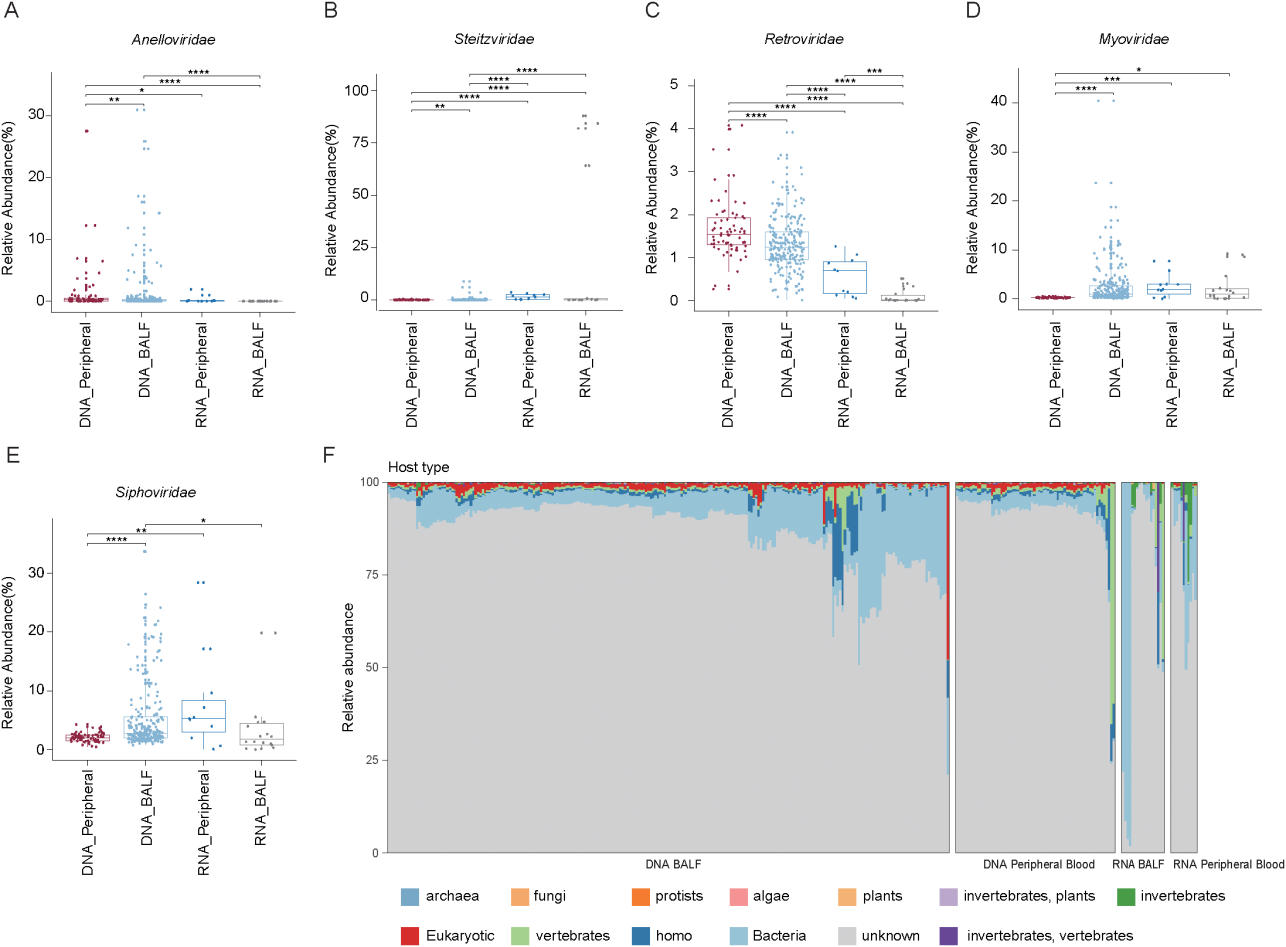
Analysis of the relative abundance of major viral families and the predicted host distribution in BALF and peripheral blood samples using DNA and RNA sequencing methods. **(A-E)** Box plot showing the relative abundance of the five virome families in four groups. Wilcoxon rank sum test: *P < 0.05; **P < 0.01, ***P < 0.001, ****P < 0.0001. **(F)** Bar graph showing the host origin of viruses in BALF and peripheral blood samples.

At the family level, only 13.6% of total viral abundance could be assigned to known viral families. The most abundant families were *Siphoviridae* (average relative abundance: 4.6 ± 5.3%) and *Myoviridae* (2.0 ± 3.7%), followed by *Retroviridae* (1.3 ± 0.7%), *Steitzviridae* (1.1 ± 8.7%), and *Anelloviridae* (1.1 ± 3.7%) (**Figure 1E**). Among these, *Retroviridae* exhibited a significant increase in the RNA-blood group compared to the RNA-BALF group. Conversely, *Anelloviridae*, *Steitzviridae*, *Myoviridae*, and *Siphoviridae* were significantly more enriched in DNA-BALF than in DNA-blood samples, whereas *Retroviridae* was less abundant in BALF (**Figures 2A–E**). These observations underscore distinct differences in virome composition between pulmonary and blood compartments. Regarding host prediction results for the virome, only 13.61% of viruses had their hosts successfully predicted. Bacteria (average relative abundance 8.8 ± 11.9% in all samples) were the predominant hosts, followed by homo (1.5 ± 3.4%), vertebrates (1.5 ± 5.6%), and eukaryotic (1.3 ± 2.7%) (**Figure 2F**). These results further reflect the bacterial dominance and diverse host associations of the human virome in clinical samples.

### Differences of the virome between BALF and blood samples

**Figure 3:**
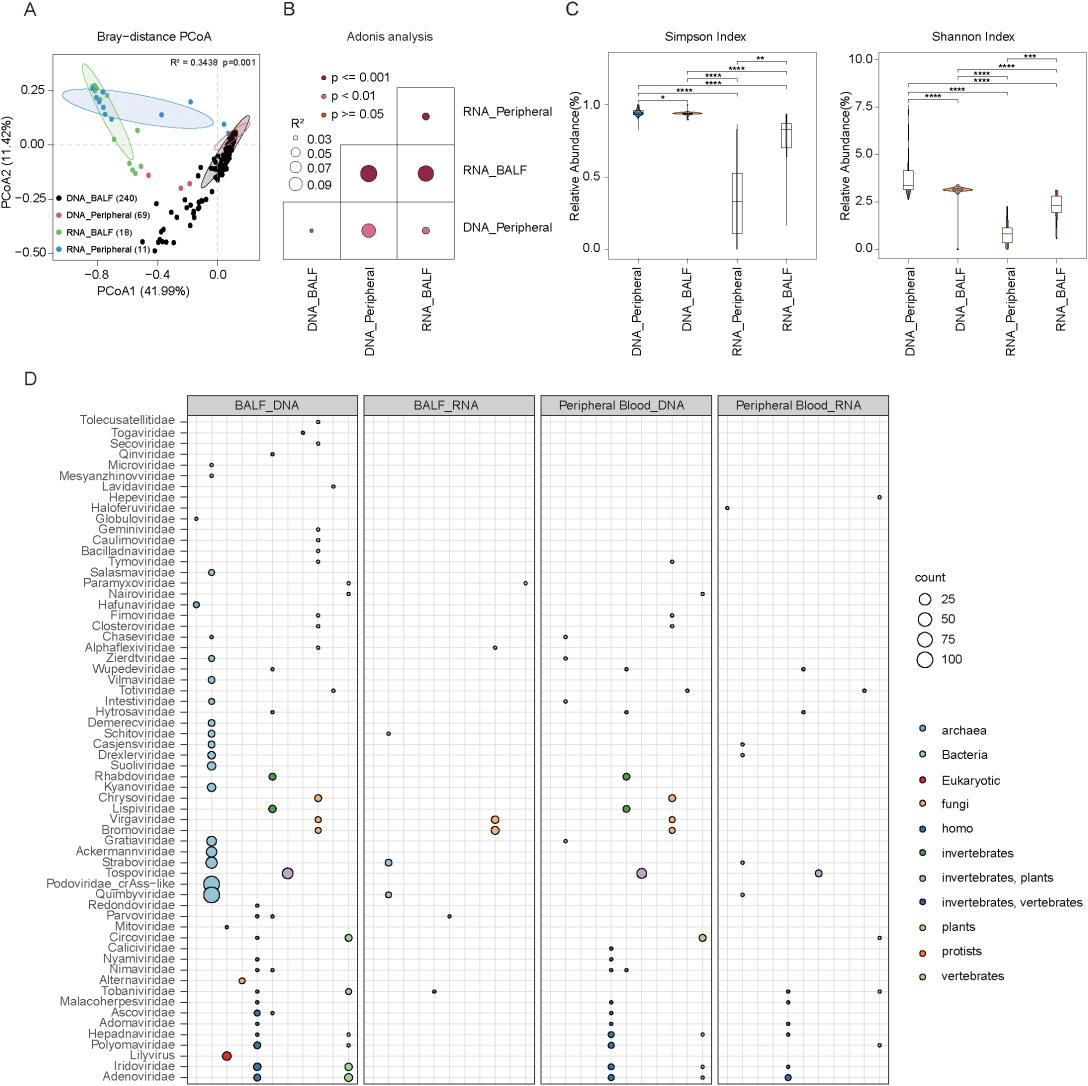
Comparative analysis of the virome composition in peripheral blood and BALF samples using DNA and RNA sequencing methods. **(A)** Scatter plots showing beta diversity, representing the compositional variation of the virus across all groups. Samples were plotted along the first and second principal coordinates (PCoA1 and PCoA2) with the associated explained variance ratios of these coordinates. **(B)** PERMANOVA analysis of the BALF and peripheral blood microbial results of each group, darker points indicate larger p values, while larger circles correspond to larger R^2^ values. **(C)** Boxplots depict virus Shannon and simpson index for all groups, respectively. Significance levels were determined using the Wilcoxon rank-sum test (*P < 0.05; **P < 0.01, ***P < 0.001, ****P < 0.0001). **(D)** The circle diagram shows the predicted distribution of viruses and their hosts in BALF and peripheral blood samples of DNA and RNA sequencing. The size of the circle represents the number of viruses, and different colors represent different host sources.

To investigate the differences in virome composition between BALF and peripheral blood samples, we assessed both within-group and between-group diversity using alpha and beta diversity indices. PCoA based on Bray–Curtis distances of vOTU-level profiles revealed clear separation between blood and BALF samples, with the first two principal coordinates explaining 53% of the total variance (**Figure 3A**). This distinction was further supported by PERMANOVA, confirming significant differences in overall virome composition between the two sample types (**Figure 3B**). To further examine differences in viral richness and evenness, we calculated alpha diversity metrics using the Shannon and Simpson indices. Comparative analysis using the Wilcoxon rank-sum tests demonstrated that in DNA-sequenced samples, BALF harbored significantly higher viral richness compared to blood samples. In contrast, in RNA-sequenced samples, BALF exhibited significantly lower richness than blood (P < 0.05) (**Figure 3C**), indicating that sample type and sequencing strategy both influence observed virome diversity.

We also examined the predicted host sources of the virome across different groups. In DNA-sequenced BALF samples, viruses were predominantly associated with bacterial hosts, followed by *Homo sapiens*, plants, and invertebrates. Conversely, DNA-sequenced blood samples showed a host profile dominated by *Homo sapiens*, with lower bacterial representation and notable contributions from plant and invertebrate hosts. RNA-sequenced BALF samples were also primarily associated with bacterial hosts, though to a lesser extent than their DNA-sequenced counterparts. Additional hosts included plants, invertebrates, and vertebrates. Viral families such as *Straboviridae* and *Quimbyviridae*, known to infect bacteria, were present at low abundance. In RNA-sequenced blood samples, viral hosts were mainly *Homo sapiens* and vertebrates, with bacterial contributions notably reduce. These results collectively suggest that DNA-sequenced BALF viromes are largely shaped by bacterial hosts, while blood viromes—particularly those captured by RNA sequencing—exhibit broader host diversity, including a higher proportion of eukaryotic hosts. This underscores distinct ecological niches and host–virus interactions across sample types and sequencing modalities (**Figure 3D**).

### Impact of disease type and clinical outcomes on the virome

**Figure 4:**
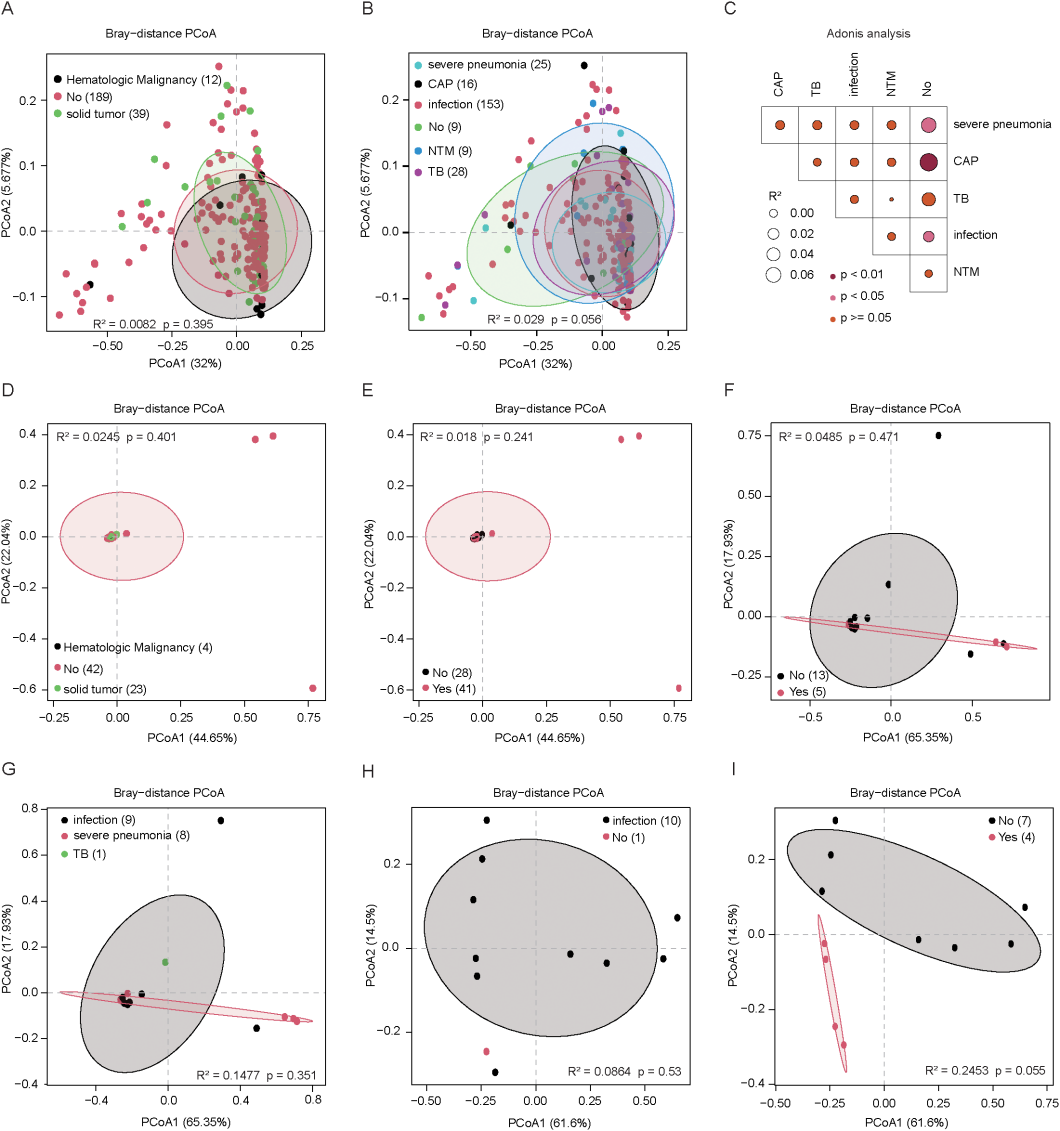
The virome composition in peripheral blood and BALF samples of different disease types was analyzed using DNA and RNA sequencing methods, each node represents a sample. **(A)** The scatter plot of *β*-diversity shows the compositional differences in the BALF virome of DNA-sequenced samples from patients with different types of tumors. **(B)** The scatter plot of *β*-diversity shows the compositional differences in the BALF virome of DNA-sequenced samples from patients with different types of pulmonary disease. **(C)** PERMANOVA analysis the compositional differences in the BALF virome of DNA-sequenced samples from patients with different types of pulmonary disease, darker points indicate larger p-values, while larger circles correspond to larger R^2^ values. **(D)** The scatter plot of *β*-diversity shows the compositional differences in the peripheral blood virome of DNA-sequenced samples from patients with different types of tumors. **(E)**. The scatter plot of *β*-diversity shows the compositional differences in the peripheral blood virome of DNA-sequenced samples from patients with different types of pulmonary disease. **(F)** The scatter plot of *β*-diversity shows the compositional differences in the BALF virome of RNA-sequenced samples from patients with different types of tumors. **(G)** The scatter plot of *β*-diversity shows the compositional differences in the BALF virome of RNA-sequenced samples from patients with different types of pulmonary disease. **(H)** The scatter plot of *β*-diversity shows the compositional differences in the peripheral blood virome of RNA-sequenced samples from patients with different types of pulmonary disease. **(I)** The scatter plot of *β*-diversity shows the compositional differences in the peripheral blood virome of RNA-sequenced samples from patients with different types of tumors.

To evaluate the influence of disease type on virome composition, we first conducted PCoA. In DNA-sequenced samples, the presence of tumors did not significantly alter the virome profile in BALF (**Figure 4A**). In contrast, patients with pulmonary diseases—such as community-acquired pneumonia (CAP), general pulmonary infections, and severe pneumonia—exhibited distinct pulmonary virome compositions compared to those without pulmonary involvement (**Figures 4B, C**). In the CAP samples, a substantial proportion (92.8%) of the total abundance was attributed to unknown viral families. Among the annotated viruses, *Siphoviridae* were most abundant (3.05 ± 2.42%), followed by *Retroviridae* (3.05 ± 2.42%), *Myoviridae* (1.67 ± 0.92%), and *Autographiviridae* (0.67 ± 0.44%). In patients with other pulmonary infections, known viral families accounted for 12.85% of the total abundance, with *Siphoviridae* (5.53 ± 5.65%) remaining dominant, followed by *Myoviridae* (2.48 ± 3.49%), *Retroviridae* (1.28 ± 0.58%), and *Anelloviridae* (1.16 ± 3.95%). In severe pneumonia cases, 16.57% of the virome could be annotated, with *Herpesviridae* (4.16 ± 11.89%) and *Siphoviridae* (4.00 ± 4.52%) being the most enriched, followed by *Anelloviridae* (2.78 ± 5.93%), *Retroviridae* (1.34 ± 0.54%), and *Myoviridae* (1.20 ± 1.95%). In contrast, no significant virome differences were observed in blood samples regardless of tumor presence or pulmonary disease (**Figures 4D, E**). Similarly, RNA-sequenced samples from both BALF and blood showed no notable virome differences across disease types (**Figures 4F-I**), indicating that disease-related alterations are more pronounced in DNA-sequenced pulmonary samples.

**Figure 5:**
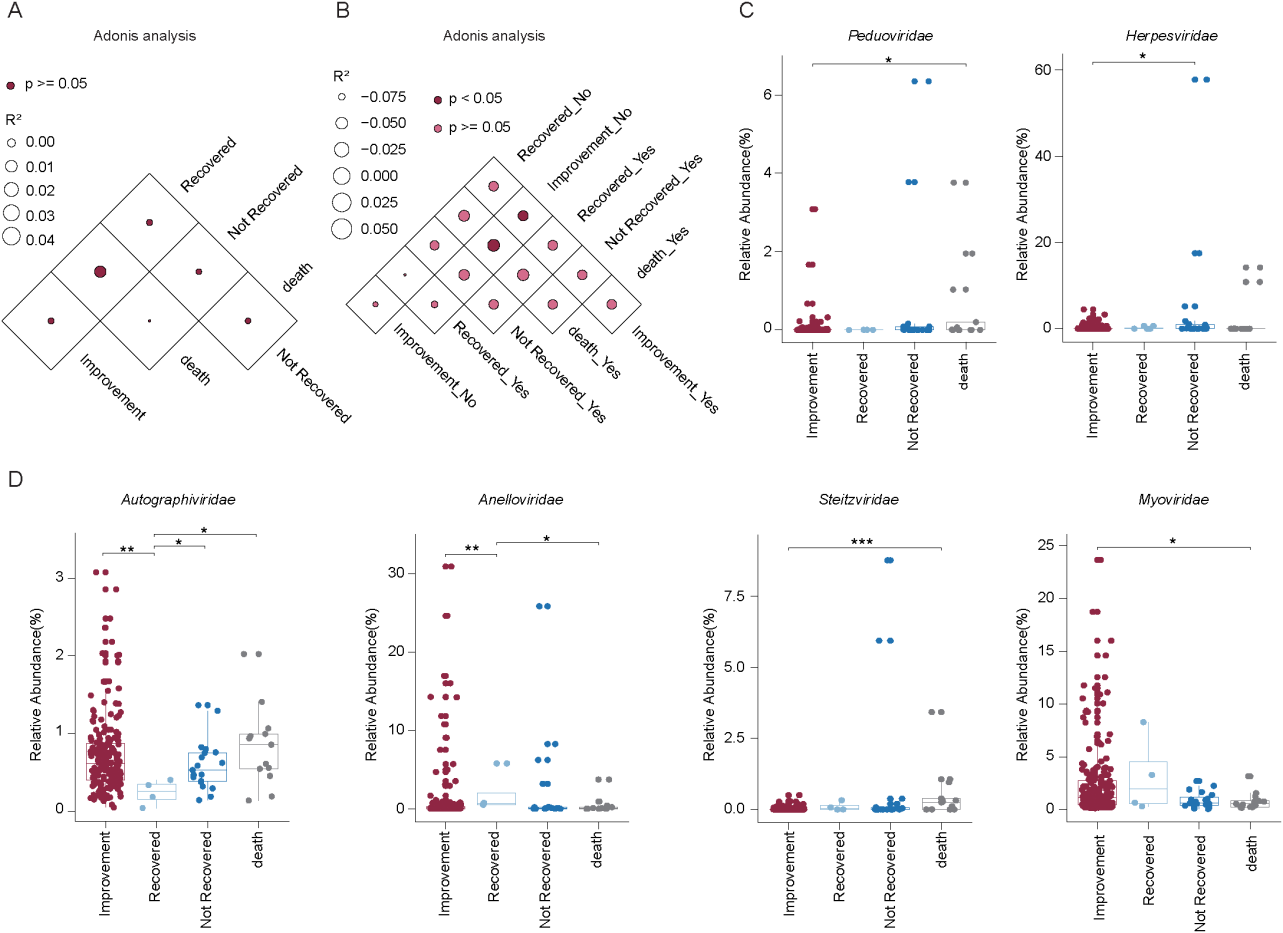
Impact of medical intervention on viral composition in BALF samples from patients with varying clinical outcomes. **(A)** PERMANOVA showing viral composition in BALF samples across different clinical outcomes. Darker points indicate larger p values, while larger circles correspond to larger R^2^ values. **(B)** PERMANOVA analysis comparing viral composition in BALF samples grouped by infection status (pulmonary infection vs. non-infection) and clinical outcomes. Darker points indicate larger p values, while larger circles correspond to larger R^2^ values. **(C-D)** Box plots showing the relative abundance of six viral families across four clinical outcome groups in pulmonary infection samples. Significant differences were assessed using the Wilcoxon rank sum test, with significance levels indicated as follows: *P< 0.05; **P < 0.01; ***P < 0.001.

To further investigate the impact of clinical outcomes, we analyzed DNA-sequenced BALF samples from patients with different treatment results (death, non-recovery, improvement, recovery). No statistically significant differences in overall virome composition were observed across these outcome groups (*P* > 0.05) (**Figure 5A**). However, given the heterogeneous sample sizes across pulmonary infection subtypes and the presence of co-infections and complications, we reclassified the BALF samples into two broader categories: pulmonary infection and non-infection. We then calculated the variance explained by viral composition for both clinical outcomes and infection status. Significant differences (*P* < 0.05) existed only between samples from patients with improved outcomes and non-infection, and those from patients with infection who either died or showed improvement (**Figure 5B**). Moreover, within the pulmonary infection group, we identified notable variations in virome composition at the viral family level across different clinical outcomes (**Figures 5C, D**).

Collectively, these findings demonstrate that pulmonary diseases, but not tumors, have a substantial impact on the virome composition of DNA-sequenced BALF samples. No such effects were observed in blood samples or RNA-sequenced datasets. This highlights the importance of considering disease context—particularly pulmonary conditions—when analyzing virome dynamics across different biological compartments and sequencing strategies.

### Relationships between viruses and bacteria in pulmonary samples

**Figure 6:**
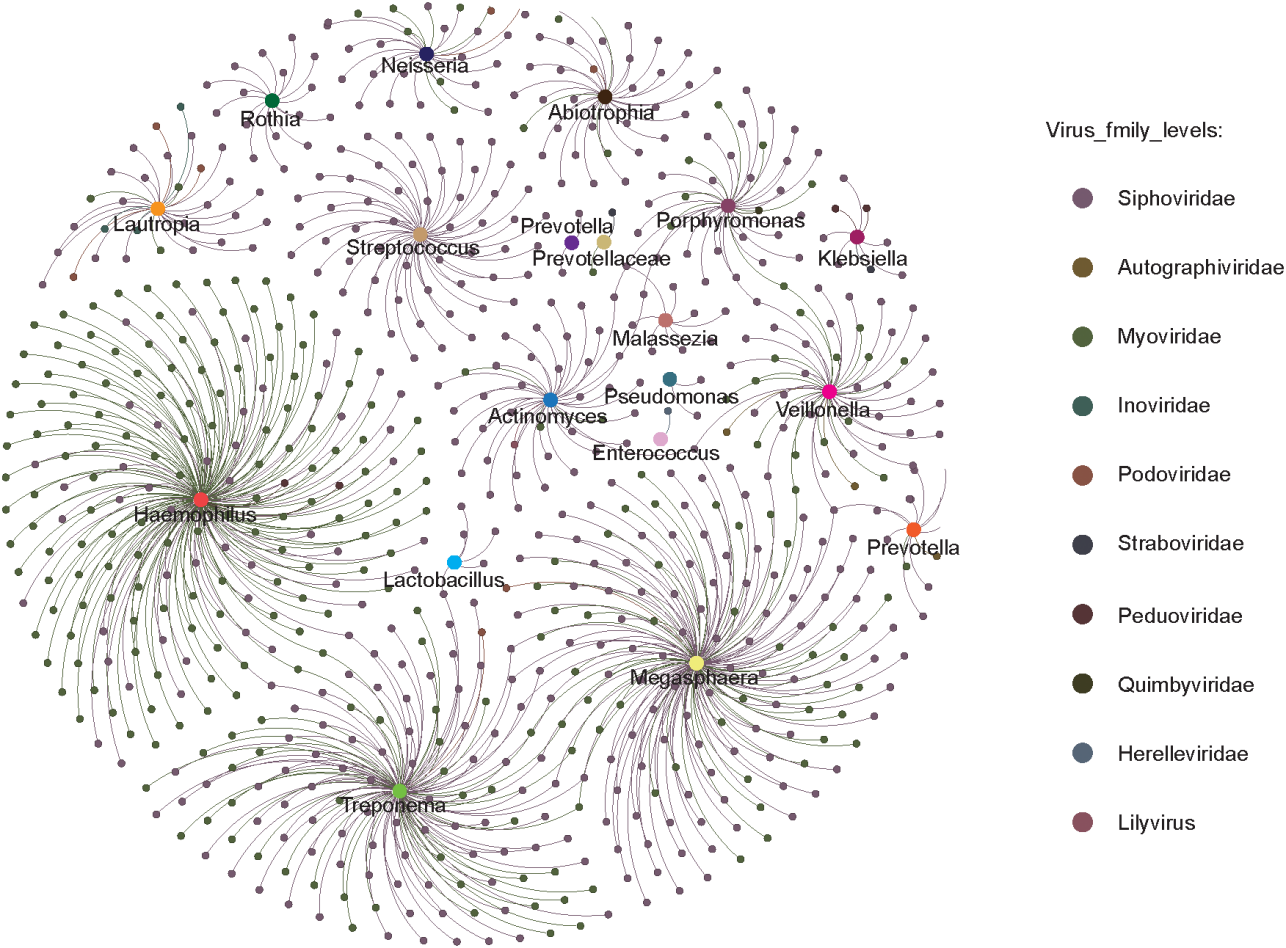
The circles represent bacteria and viruses, respectively, with larger circles representing bacteria and smaller circles representing viruses; the colors represent their taxonomic assignment at the genus (bacteria) or family (virus) level.

To elucidate the interactions between viruses and bacteria in the pulmonary environment, we conducted a Spearman correlation analysis between vOTUs and 67 bacterial genera. In BALF samples, this analysis identified 162 statistically significant associations (Spearman’s correlation coefficient > 0.6, p < 0.05), which were visualized as a virus–bacteria correlation network. Among the viral families, *Siphoviridae* and *Myoviridae* displayed the highest number of associations. *Siphoviridae* were predominantly correlated with *Haemophilus*, *Megasphaera*, *Treponema*, and *Veillonella*, while *Myoviridae* were primarily linked to *Streptococcus*, *Prevotella*, *Actinomyces*, and *Lautropia*. These patterns suggest that these bacterial genera may serve as principal hosts for the respective viral families. Other viral families, such as *Podoviridae, Peduoviridae*, *Autographiviridae*, and *Inoviridae*, showed more limited host associations, mainly with *Pseudomonas*, *Neisseria*, *Klebsiella*, and *Prevotellaceae*. Notably, certain bacterial genera like *Streptococcus*, *Veillonella*, and *Prevotella* were connected to multiple viral families, indicating their high susceptibility to viral colonization and their potentially central role in the virus–host network within the pulmonary microbiome (**Figure 6**). These findings underscore that specific bacterial taxa may act as keystone hosts within the pulmonary virome, facilitating diverse viral interactions. However, it is important to note that these associations are based on correlation and do not necessarily indicate direct virus–host interactions. Nevertheless, the extensive connectivity between viral and bacterial taxa highlights the necessity of integrating virome and bacteriome data when investigating pulmonary infections. This network-based perspective provides valuable insights into the ecological complexity of pulmonary microbial communities and may help identify candidate microbial targets for therapeutic intervention and further studies on viral–bacterial co-infection mechanisms.

## Discussion

This comprehensive mNGS-based investigation reveals distinct and clinically relevant features of the virome in bronchoalveolar lavage fluid (BALF) and peripheral blood. Our findings demonstrate that anatomical compartment, sequencing strategy, and disease context are major determinants of viral community structure, with important implications for diagnostics and host–microbe interactions.

We observed striking differences in virome composition between BALF and blood. In BALF, bacteriophages—particularly *Siphoviridae* and *Myoviridae*—were dominant, consistent with prior reports linking phages to the densely colonized respiratory microbiota (Manrique et al., 2016; Kaelin et al., 2022). Their abundance suggests a critical role in shaping bacterial communities, potentially affecting the onset and progression of pulmonary infections (Pride et al., 2012; Abbas et al., 2019). In contrast, the blood virome was enriched for human-associated viruses such as *Retroviridae* and *Anelloviridae*, which are known to establish systemic infections and persist in circulation (Nakamura et al., 2009; Li et al., 2013). This compartmental specificity highlights distinct ecological niches and transmission routes of viruses within the human body.

Sequencing strategy markedly influenced virome profiles. DNA sequencing captured more diverse and phage-rich viromes in BALF, whereas RNA sequencing detected a broader range of eukaryotic viruses, particularly in blood. These differences likely reflect the inherent biases of each method—DNA sequencing favoring DNA viruses (especially phages), and RNA sequencing capturing active RNA viruses—underscoring the importance of combined approaches for virome surveillance.

Disease status had a notable impact on the pulmonary virome, particularly in patients with community-acquired pneumonia (CAP) and severe pneumonia. These samples showed higher proportions of unclassified viruses, indicating the presence of novel or under-characterized viral taxa and revealing the limitations of current reference databases. *Siphoviridae* remained consistently enriched across disease types, suggesting a potential role in disease pathogenesis through phage–bacteria interactions (Wylie et al., 2012; Moustafa et al., 2017). In contrast, tumor status had minimal influence on virome composition, and blood viromes were less responsive to disease status overall. These findings emphasize the need to consider clinical context, especially infectious disease status, in virome interpretation.

The virus–bacteria correlation network in BALF revealed intricate associations, particularly between dominant phages and bacterial genera such as *Haemophilus*, *Streptococcus*, and *Veillonella*. These interactions suggest a central role for phages in modulating respiratory bacterial communities and highlight the ecological complexity of the pulmonary microbiome. Although correlation does not confirm causation, the observed patterns provide a foundation for future mechanistic studies on viral-bacterial co-dynamics (Lim et al., 2014).

Finally, our findings underscore the diagnostic value of mNGS in respiratory and systemic infections. The ability to detect both known and novel viruses from BALF and blood samples enables better differentiation between viral and bacterial infections, guiding targeted antimicrobial use. Moreover, blood virome profiling in immunocompromised patients may support early detection of opportunistic infections, improving patient outcomes (Van Rijn et al., 2019).

## Conclusion

In conclusion, this study highlights the utility of mNGS in uncovering virome complexity across body compartments. The findings underscore the compartment-specific nature of the virome, its modulation by disease, and its interactions with bacterial hosts. Future work should aim to characterize the functional roles of these viruses, assess their impact on host immunity and microbial communities, and track virome dynamics longitudinally. Integrating viromics with metatranscriptomics, metaproteomics, and host-response data will be essential to fully understand the role of the virome in health and disease.

## Data Availability

The datasets presented in this study can be found in online repositories. The names of the repository/repositories and accession number(s) can be found in the article/**Supplementary Material**.
